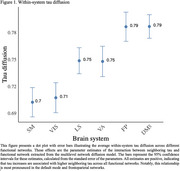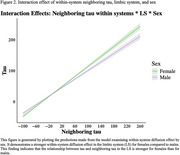# Sex Differences in the Diffusion of Tau Neurofibrillary Tangles Across Functional Brain Networks

**DOI:** 10.1002/alz70855_100019

**Published:** 2025-12-23

**Authors:** Plamena P. Powla, Theyaneshwaran Jayaprakash, Connor Lee Cornelison, Tracy Sweet, Li Shen, Sujuan Gao, Meichen Yu, Yu‐Chien Wu, Yize Zhao, Andrew J. Saykin, Selena Wang

**Affiliations:** ^1^ Indiana University School of Medicine, Indianapolis, IN, USA; ^2^ Indiana University, Bloomington, IN, USA; ^3^ University of Maryland, College Park, MD, USA; ^4^ University of Pennsylvania, Philadelphia, PA, USA; ^5^ School of Medicine, Indiana University‐Purdue University at Indianapolis, Indianapolis, IN, USA; ^6^ Indiana University School of Medicine, Department of Radiology and Imaging Sciences, Indianapolis, IN, USA; ^7^ Yale University, New Haven, CT, USA

## Abstract

**Background:**

Alzheimer's disease is characterized by the accumulation and spread of tau neurofibrillary tangles across cortical regions, driving cognitive decline. However, many cortical regions exhibit little tau, despite their functional connections to regions with high levels. In fact, recent evidence links elevated tau to hypoconnectivity in the default mode network (DMN) during early phases of disease, suggesting possible differential tau diffusion across functional networks. Therefore, the aim of this study is to examine tau diffusion (spreading of tau across functionally connected regions) and whether this is differentiated by functional network. To do this, novel multilevel network diffusion models are proposed to compare diffusion across functional networks and examine whether this differs by sex.

**Method:**

Included are 321 subjects from the third phase of the Alzheimer's Disease Neuroimaging Initiative (ADNI 3). Multilevel network diffusion models, first proposed by Frank et al. as social influence models to study the dynamics of teacher interactions, were adapted to examine diffusion. Diffusion is defined as the spreading of tau via neighboring (functionally connected) regions. Functional networks include DMN, limbic system (LSN), frontoparietal (FPN), dorsal attention (DAN), sensorimotor (SMN), visual (VISN), and ventral attention (VAN), which were parcellated according to the Desikan‐Killiany Atlas. We compared diffusion across these networks and investigated its interactions with sex.

**Result:**

Analyses reveal significant diffusion differences within DAN (Estimate=.243, *p* <.001), LSN (Estimate=‐.038, *p* <.001), SMN (Estimate=.084, *p* <.001), VAN (Estimate=‐.039, *p* = .004), and VISN (Estimate=‐.079, *p* <.001) compared to the DMN. Figure 1 illustrates the rate of diffusion across networks with highest diffusion found in FPN and DMN, and lowest in SMN and VISN. Additionally, we found sex differences in LSN diffusion (Estimate=.047, *p* = .037; see Figure 2), with stronger diffusion in females, suggesting that tau spreads faster in females than males. These findings are based on two models: one for diffusion differences across networks and another for sex‐based diffusion differences across networks.

**Conclusion:**

This study represents an in‐depth investigation of tau diffusion, showing highest diffusion in the FPN and DMN, consistent with previous research on network‐specific glucose metabolism. Additionally, higher tau diffusion in the LSN of females supports prior studies showing greater tau distribution in limbic regions of women.